# Modern subcutaneous implantable defibrillator therapy in patients with cardiomyopathies and channelopathies: data from a large multicentre registry

**DOI:** 10.1093/europace/euad239

**Published:** 2023-08-03

**Authors:** Federico Migliore, Mauro Biffi, Stefano Viani, Raimondo Pittorru, Pietro Francia, Paolo Pieragnoli, Paolo De Filippo, Giovanni Bisignani, Gerardo Nigro, Antonio Dello Russo, Ennio Pisanò, Pietro Palmisano, Antonio Rapacciuolo, Massimo Stefano Silvetti, Carlo Lavalle, Antonio Curcio, Roberto Rordorf, Mariolina Lovecchio, Sergio Valsecchi, Antonio D’Onofrio, Giovanni Luca Botto

**Affiliations:** Department of Cardiac, Thoracic Vascular Sciences and Public Health, University of Padova, Padova, Italy; Institute of Cardiology, Department of Experimental, Diagnostic and Specialty Medicine, University of Bologna, Policlinico S.Orsola-Malpighi, Bologna, Italy; Second Cardiology Division, Cardio-Thoracic and Vascular Department, University Hospital of Pisa, Pisa, Italy; Department of Cardiac, Thoracic Vascular Sciences and Public Health, University of Padova, Padova, Italy; Cardiology, Department of Clinical and Molecular Medicine, Sant’Andrea Hospital, University Sapienza, Rome, Italy; Arrhythmic Disease Unit, University of Florence, Florence, Italy; Cardiac Electrophysiology and Pacing Unit, Papa Giovanni XXIII Hospital, Bergamo, Italy; Division of Cardiology, Castrovillari Hospital, Cosenza, Italy; Department of Translational Medical Sciences, University of Campania ‘Luigi Vanvitelli’, Naples, Italy; Cardiology and Arrhythmology Clinic, Università Politecnica delle Marche, Ancona, Italy; Cardiology Unit, ‘Vito Fazzi’ Hospital, Lecce, Italy; Cardiology Unit, ‘Card. G. Panico’ Hospital, Tricase, Italy; Department of Advanced Biomedical Sciences, Federico II University of Naples, Naples, Italy; Pediatric Cardiology and Cardiac Arrhythmia/Syncope Unit, Bambino Gesù Children’s Hospital IRCCS, Rome, Italy; Cardiology Department, Policlinico Umberto I - La Sapienza University, Rome, Italy; Dipartimento di Scienze Mediche e Chirurgiche, Università degli Studi Magna Graecia, Campus di Germaneto, Catanzaro, Italy; Cardiac Intensive Care Unit, Arrhythmia and Electrophysiology and Experimental Cardiology, IRCCS Fondazione Policlinico S. Matteo, Pavia, Italy; Cardiac Rhythm Management Division, Boston Scientific, Milan, Italy; Cardiac Rhythm Management Division, Boston Scientific, Milan, Italy; ‘Unità Operativa di Elettrofisiologia, Studio e Terapia delle Aritmie’, Monaldi Hospital, Naples, Italy; Department of Clinical cardiology and Electrophysiology ASST Rhodense, Rho and Garbagnate M.se, Milan, Italy

**Keywords:** Implantable defibrillator, Subcutaneous, Inappropriate shock, Cardiomyopathies, Channelopathies

## Abstract

**Aims:**

Patients with cardiomyopathies and channelopathies are usually younger and have a predominantly arrhythmia-related prognosis; they have nearly normal life expectancy thanks to the protection against sudden cardiac death provided by the implantable cardioverter defibrillator (ICD). The subcutaneous ICD (S-ICD) is an effective alternative to the transvenous ICD and has evolved over the years. This study aimed to evaluate the rate of inappropriate shocks (IS), appropriate therapies, and device-related complications in patients with cardiomyopathies and channelopathies who underwent modern S-ICD implantation.

**Methods and results:**

We enrolled consecutive patients with cardiomyopathies and channelopathies who had undergone implantation of a modern S-ICD from January 2016 to December 2020 and who were followed up until December 2022. A total of 1338 S-ICD implantations were performed within the observation period. Of these patients, 628 had cardiomyopathies or channelopathies. The rate of IS at 12 months was 4.6% [95% confidence interval (CI): 2.8–6.9] in patients with cardiomyopathies and 1.1% (95% CI: 0.1–3.8) in patients with channelopathies (*P* = 0.032). No significant differences were noted over a median follow-up of 43 months [hazard ratio (HR): 0.76; 95% CI: 0.45–1.31; *P* = 0.351]. The rate of appropriate shocks at 12 months was 2.3% (95% CI: 1.1–4.1) in patients with cardiomyopathies and 2.1% (95% CI: 0.6–5.3) in patients with channelopathies (*P* = 1.0). The rate of device-related complications was 0.9% (95% CI: 0.3–2.3) and 3.2% (95% CI: 1.2–6.8), respectively (*P* = 0.074). No significant differences were noted over the entire follow-up. The need for pacing was low, occurring in 0.8% of patients.

**Conclusion:**

Modern S-ICDs may be a valuable alternative to transvenous ICDs in patients with cardiomyopathies and channelopathies. Our findings suggest that modern S-ICD therapy carries a low rate of IS.

**Clinical Trial Registration:**

URL: http://clinicaltrials.gov/Identifier: NCT02275637.

## Graphical Abstract

**Graphical Abstract:**
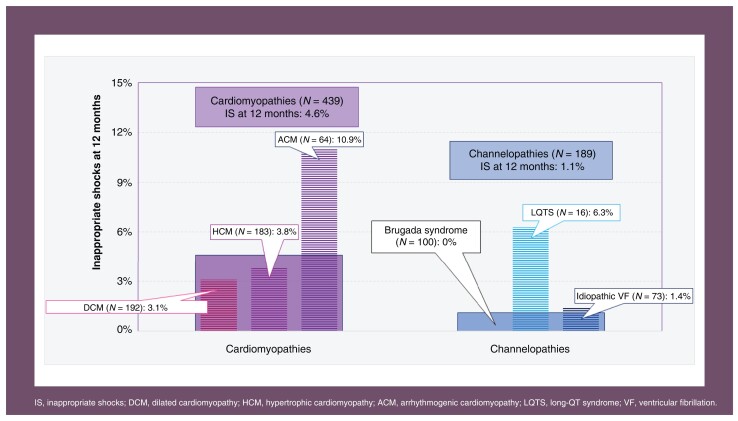

**What’s new?**

- The rate of inappropriate shocks at 12 months is higher in patients with cardiomyopathies than in those with channelopathies (4.6% vs. 1.1%), despite similar values over the long-term follow-up.
- Device-related complications at 12 months are reported in 0.9% of patients with cardiomyopathies and 3.2% of those with channelopathies. The management of all device-related complications requiring surgical intervention is safe and effective.
- Over a long-term follow-up, the need for pacing is low, occurring in 0.8% of the overall population.

## Introduction

Life-threatening ventricular arrhythmias (VA) and sudden cardiac death (SCD) may be related to cardiomyopathies and channelopathies. In patients with cardiomyopathies and channelopathies who are at risk of SCD, the implantable cardioverter defibrillator (ICD) provides the most effective life-saving therapy.^1–10^ Patients with cardiomyopathies and channelopathies are usually younger and have a predominantly arrhythmia-related prognosis; they may therefore survive for many decades and have nearly normal life expectancy thanks to the protection against SCD provided by the ICD.^1–10^ However, the improvement of survival due to ICD therapy is associated with a significant rate of inappropriate shocks (IS) and lead-related complications, which are higher than in the general ICD population.^10,11^ The subcutaneous ICD (S-ICD) is now recognized to be an effective alternative to the transvenous ICD (TV-ICD) for the prevention of SCD among high-risk patients who do not need pacing or cardiac resynchronization therapy.^12–14^ Indeed, the S-ICD, in addition to displaying the same efficacy in interrupting life-threatening VA, enables the risk of systemic infection and lead failure to be reduced, which are the most common complications of TV-ICD and often require surgical revision.^12,14^ Early studies on the S-ICD demonstrated the effectiveness of the first-generation devices, despite relatively high IS rates. However, S-ICD therapy has evolved over the years, and more recent data show that the use of high-rate cut-off, current-generation electrogram filtering and discrimination algorithms have significantly reduced IS rates;^15–19^ with modern devices, the reported rate is 2.4%/year, a figure even lower than that of TV-ICD with modern programming.^13^ However, few studies have been conducted on the performance of S-ICD in patients with cardiomyopathies and channelopathies.^20–26^ Because of predisposing electrocardiogram (ECG) depolarization/repolarization changes, these patients have a potentially increased risk of double QRS counting or P- and/or T-wave oversensing (TWO), potentially inducing IS delivery. Long-term data on modern S-ICD and the type of implantation technique in patients with cardiomyopathies and channelopathies are lacking. Thus, the aim of this multicentre study was to evaluate the rate of IS, appropriate therapies, and device-related complications during long-term follow-up in patients with cardiomyopathies and channelopathies who undergo modern S-ICD implantation.

## Methods

### Study population

The present study was a retrospective analysis of data collected within the framework of the prospective ‘Rhythm Detect’ registry. The study population consisted of consecutive patients with cardiomyopathies and channelopathies who had undergone *de novo* implantation of a modern S-ICD (EMBLEM S-ICD, Model A209/A219, Boston Scientific Inc., Natick, MA, USA), at 66 Italian centres (see Appendix) from January 2016 to December 2020, and who were followed up until December 2022. For the aim of this analysis, we excluded old-generation pulse generators (SQ-RX, Model 1010), which were not equipped with the SMART Pass filter, and patients with a follow-up period shorter than 2 years. Cardiomyopathies and channelopathies were defined as either structured cardiomyopathies, including dilated cardiomyopathy, hypertrophic cardiomyopathy (HCM), and arrhythmogenic cardiomyopathy (ACM), or channelopathies including Brugada syndrome (BrS), long-QT syndrome (LQTS), and idiopathic ventricular fibrillation (IVF). Cardiomyopathies and channelopathies were diagnosed according to general guidelines and/or available task force consensus statements.^7–9,27–29^ Baseline clinical characteristics, electrocardiographic data, indication for implantation, ECG screening, and device parameters were collected. The registry was ethically approved by the host institutions and was conducted according to the Helsinki declaration. All patients provided written informed consent for data storage and analysis.

### S-ICD implantation technique, defibrillation testing, and device programming

Before implantation, all patients were screened for eligibility for S-ICD by means of the Boston Scientific screening tool, which is based on the surface ECG limb lead recording over the left and/or right parasternal regions to simulate the three S-ICD sensing vectors. To be eligible for S-ICD implantation, at least one vector had to pass the test in both the erect and supine postures. Implantation was performed in an electrophysiology laboratory under standard sterile conditions and general anaesthesia, local anaesthesia with conscious sedation, or ultrasound-guided serratus anterior plane block, as previously reported.^30^ Antibiotic prophylaxis was administered 1 h before the procedure. All S-ICD implantations were performed by experienced operators. According to physician preference, the pulse generator was positioned in a subcutaneous pocket or in an intermuscular position (between the serratus anterior and the latissimus dorsi muscles) as previously reported in detail.^31,32^ For lead deployment, physicians adopted the three-incision technique, i.e. pocket incision, xiphoid incision, and superior incision at the sternomanubrial junction, or the two-incision technique, i.e. the superior incision is avoided by positioning the lead by means of a peel-away sheath introducer.^31,32^ The position of the lead and pulse generator relative to the heart silhouette was checked by means of fluoroscopy. At the end of the procedure, the decision to perform defibrillation testing (DT) was left to the discretion of the implanting physician. Defibrillation testing was considered successful if the device detected and terminated the induced ventricular fibrillation (VF) by using ≤ 65 J shock energy. Programming of the parameters for the detection of ventricular tachycardia (VT) or VF was also left to the discretion of the implanting centre. Physicians were free to set parameters on hospital discharge and adjust them during follow-up, in order to fit the specific characteristics of the patient, and on the basis of the best available evidence. The sensing vector (primary, secondary, or alternate) was automatically selected by the device at the time of implantation and optimized during supine and upright positions before discharge. After implantation, patients were followed up in accordance with the standard practice of the participating centres.

### Definition of outcomes

The primary endpoint of the study was the rate of IS. An S-ICD shock was classified as inappropriate when it was delivered for any rhythm other than VF or VT, including supraventricular arrhythmias, cardiac/non-cardiac oversensing, or device or lead malfunction. Secondary endpoints consisted of appropriate shocks and device-related complication rates. For the analysis of therapy efficacy, we reported when the first shock successfully converted the VA to sinus rhythm and the final efficacy. Complications were defined as events that led to intervention or prolongation of hospitalization and included device infection, lead repositioning or replacement, and other complications related to the lead or generator. The rates of endpoints were evaluated at 12 months, and cumulative survival rates were also measured over the entire follow-up period.

### Statistical analysis

Descriptive statistics are reported as means ± SD for normally distributed continuous variables or medians and interquartile range (25th–75th percentile) in the case of skewed distribution. Normality of distribution was tested by means of the non-parametric Kolmogorov–Smirnov test. Categorical variables are reported as percentages. Differences were compared by means of Mann–Whitney or Wilcoxon non-parametric tests for non-Gaussian variables. Differences in proportions were compared by means of a Chi-square analysis. Analysis of the cumulative survival rates was made by means of the Kaplan–Meier method, and the distributions of the groups were compared by means of a log-rank test. A *P* value <0.05 was considered significant for all tests. All statistical analyses were performed by means of R: a language and environment for statistical computing (R Foundation for Statistical Computing, Vienna, Austria).

## Results

### Study population

A total of 1338 consecutive *de novo* S-ICD implantations were performed within the observation period. Of these patients, 628 had cardiomyopathies or channelopathies: dilated cardiomyopathy (*n* = 192; 30%), HCM (*n* = 183; 29%), ACM (*n* = 64; 10%), BrS (*n* = 100; 16%), LQTS (*n* = 16; 3%), and IVF (*n* = 73; 12%). *Table 1* shows the baseline clinical characteristics and implantation variables of the overall study population and of patients stratified by disease. The S-ICD generator was positioned in a standard subcutaneous pocket in 144 (23%) patients and in an intermuscular pocket in 484 (77%); the two-incision technique was adopted in 581 (93%) procedures. On pre-discharge programming, the median conditional zone cut-off rate was 210 b.p.m. (25th–75th percentile: 200–220) and the shock zone cut-off was 230 b.p.m. (25th–75th percentile: 210–250). No major differences in device programming were found among groups (*Table 1*). Cardioversion at a shock energy of ≤ 65 J was tested in 490 (78%) patients. In patients who underwent DT, success at a shock energy of ≤ 65 J was reported in 475 (97%) cases. In one patient with BrS, successful cardioversion was not obtained with an 80 J shock and the S-ICD was not implanted.

**Table 1:** Baseline clinical characteristics and implantation variables of the overall study population and of patients stratified according to the type of underling cardiomyopathy and channelopathy

	Overall (*n* = 628)	Dilated Cardiomyopathy (*n* = 192)	HCM (*n* = 183)	ACM (*n* = 64)	BrS (*n* = 100)	LQTS (*n* = 16)	Idiopathic VF (*n* = 73)
Age, years	46 ± 15	52 ± 12	44 ± 15	38 ± 16	44 ± 12	36 ± 16	45 ± 15
Male gender, *n* (%)	480 (76)	157 (82)	143 (78)	42 (66)	80 (80)	8 (50)	50 (68)
Body mass index, kg/m^2^	26 ± 4	27 ± 5	26 ± 4	24 ± 3	25 ± 4	24 ± 4	25 ± 3
Ejection fraction, %	49 ± 16	30 ± 8	60 ± 10	53 ± 10	62 ± 5	61 ± 5	56 ± 9
Primary prevention, %	506 (81)	181 (94)	173 (94)	54 (84)	88 (88)	10 (62)	0 (0)
S-ICD in intermuscular pocket, *n* (%)	484 (77)	153 (80)	140 (77)	53 (83)	77 (77)	12 (75)	49 (67)
Two-incision technique, *n* (%)	581 (93)	182 (94)	167 (91)	61 (95)	91 (91)	14 (88)	66 (90)
Emblem generator, model A219	503 (80)	155 (81)	142 (78)	58 (91)	82 (82)	14 (87)	52 (71)
Dual-zone programming, *n* (%)	622 (99)	188 (98)	181 (99)	64 (100)	100 (100)	16 (100)	73 (100)
Conditional zone, b.p.m.	210 (200–220)	200 (200–210)	210 (200–220)	210 (200–220)	210 (200–220)	210 (200–220)	210 (200–220)
Shock zone, b.p.m.	230 (210–250)	220 (210–250)	230 (230–250)	230 (220–250)	230 (220–240)	235 (225–240)	230 (210–250)
Sensing vector:							
Primary, *n* (%)	367 (58)	121 (63)	113 (62)	39 (61)	44 (44)	12 (75)	38 (52)
Secondary, *n* (%)	217 (35)	58 (30)	52 (28)	22 (34)	52 (52)	3 (19)	30 (41)
Alternate, *n* (%)	44 (7)	13 (7)	18 (10)	3 (5)	4 (4)	1 (6)	5 (7)

HCM, hypertrophic cardiomyopathy; ACM, arrhythmogenic cardiomyopathy; BrS, Brugada syndrome; LQTS, long-QT syndrome; VF, ventricular fibrillation.

### Outcome analysis

**Figure 1:**
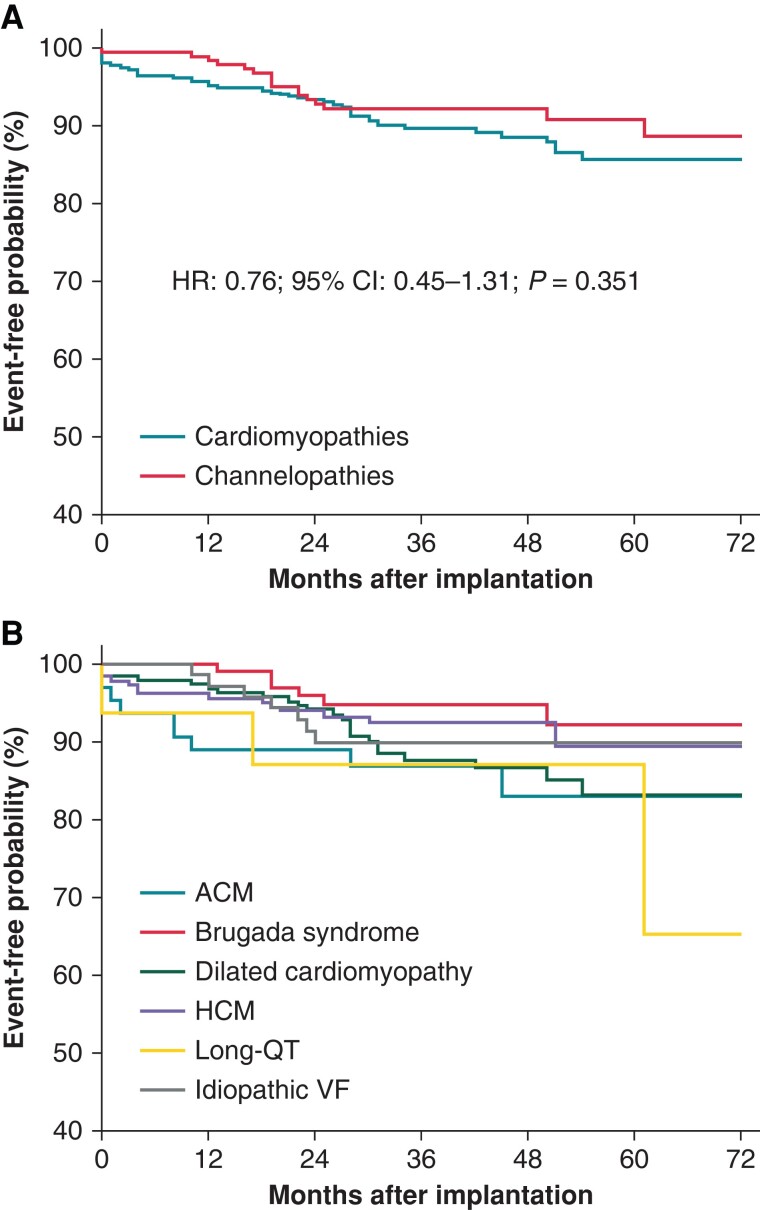
Kaplan–Meier estimates of time to first inappropriate shock, stratified by cardiomypathies and channelopathies (*A*) and type of disease (*B*). ACM, arrhythmogenic cardiomyopathy; HCM, hypertrophic cardiomyopathy; VF, ventricular fibrillation.

**Figure 2:**
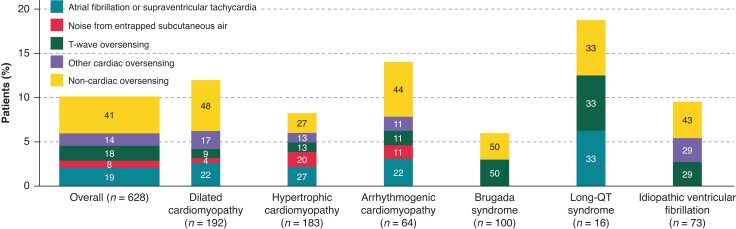
Causes of inappropriate shocks reported during follow-up and distribution according to the type of cardiomyopathy and channelopathy.

In the overall study population, over a median follow-up of 43 months (25th–75th percentile: 28–57), 21 (3%) deaths occurred. Inappropriate shocks were reported in 63 (10%) patients: 47 (11%) with cardiomyopathies and 16 (8%) with channelopathies. Specifically, IS were recorded in 23 (12%) patients with dilated cardiomyopathy, 15 (8%) with HCM, 9 (14%) with ACM, 6 (6%) with BrS, 3 (19%) with LQTS, and 7 (10%) with IVF. The rate of IS at 12 months was 3.5% [95% confidence interval (CI): 2.2–5.3] in the overall study population. The rate at 12 months was 4.6% (95% CI: 2.8–6.9) in patients with cardiomyopathies and 1.1% (95% CI: 0.1–3.8) in patients with channelopathies (*P* = 0.032). The IS rates at 12 months according to the type of disease are shown in *Table 2*. *Figure 1* shows the Kaplan–Meier analysis of time to first IS, stratified by cardiomyopathy vs. channelopathy and different types of diseases. No significant differences were noted over the entire follow-up period [hazard ratio (HR): 0.76; 95% CI: 0.45–1.31; *P* = 0.351]. The causes of IS and the distribution among different diseases are reported in *Figure 2*. The most common reason for IS was non-cardiac oversensing both in the overall study population (41%, 26 out of 63) and in all subgroups. The source of non-cardiac oversensing was myopotentials in 9 (1.4%) patients and other sources in 17 (2.7%) patients. Of the nine cases of oversensing of myopotentials, four were reported during sports activity (gym training in three cases and skiing in one case), the others during daily activities. Overall, the vast majority of IS (*n* = 52, 83%) was managed without requiring S-ICD surgical revision; 36 were solved by means of S-ICD reprogramming. In the remaining 16 cases, no reprogramming or other actions were performed; only drug therapy adjustments were reported in some cases. After the first IS, 25 out of 63 patients experienced additional episodes. The time-course of all IS (first occurrence and recurrences) reported during the observation period in patients with cardiomyopathies and channelopathies is shown in Supplementary material online, *Figure S1*.

**Table 2:** Rate of endpoints at 12 months: inappropriate shocks, appropriate shocks, and device-related complications

Twelve-month rate (95% CI)	Inappropriate shocks	Appropriate shocks	Device-related complications
Overall (*n* = 628)	3.5% (95% CI: 2.2–5.3)	2.2% (95% CI: 1.2–3.7)	1.8% (95% CI: 0.9–3.1)
Cardiomyopathies (*n* = 439)	4.6% (95% CI: 2.8– 6.9)	2.3% (95% CI: 1.1– 4.1)	0.9% (95% CI: 0.3– 2.3)
Dilated cardiomyopathy (*n* = 192)	3.1% (95% CI: 1.2–6.7)	1.6% (95% CI: 0.3–4.5)	1.0% (95% CI: 0.1–3.7)
HCM (*n* = 183)	3.8% (95% CI: 1.6–7.7)	1.6% (95% CI: 0.3–4.7)	0.5% (95% CI: 0.1–3.9)
ACM (*n* = 64)	10.9% (95% CI: 4.5–21.2)	6.3% (95% CI: 1.7–15.2)	1.6% (95% CI: 0.0–8.4)
Channelopathies (*n* = 189)	1.1% (95% CI: 0.1– 3.8)*	2.1% (95% CI: 0.6– 5.3)^#^	3.2% (95% CI: 1.2– 6.8)^$^
Brugada syndrome (*n* = 100)	0.0% (95% CI: 0.0–3.6)	0.0% (95% CI: 0.0–3.6)	3.0% (95% CI: 0.6–8.5)
Long-QT syndrome (*n* = 16)	6.3% (95% CI: 0.2–30.2)	0.0% (95% CI: 0.0–20.6)	6.3% (95% CI: 0.2–30.2)
Idiopathic VF (*n* = 73)	1.4% (95% CI: 0.0–7.4)	5.5% (95% CI: 1.5–13.4)	2.7% (95% CI: 0.3–9.5)

HCM, hypertrophic cardiomyopathy; ACM, arrhythmogenic cardiomyopathy; VF, ventricular fibrillation.

* *P* = 0.032, ^#^*P* = 1.000, and ^$^*P* = 0.074 vs. cardiomyopathies.

**Figure 3:**
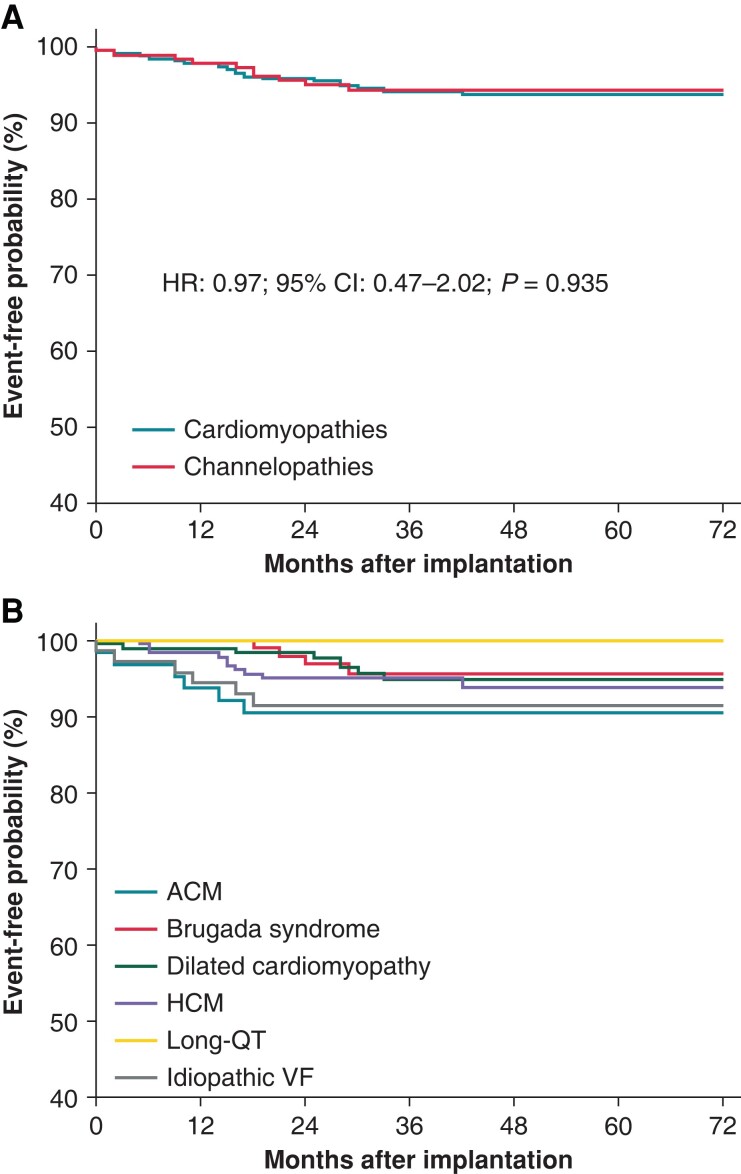
Kaplan–Meier estimates of time to first appropriate shock stratified by cardiomypathies and channelopathies (*A*) and type of disease (*B*). ACM, arrhythmogenic cardiomyopathy; HCM, hypertrophic cardiomyopathy; VF, ventricular fibrillation.

**Figure 4:**
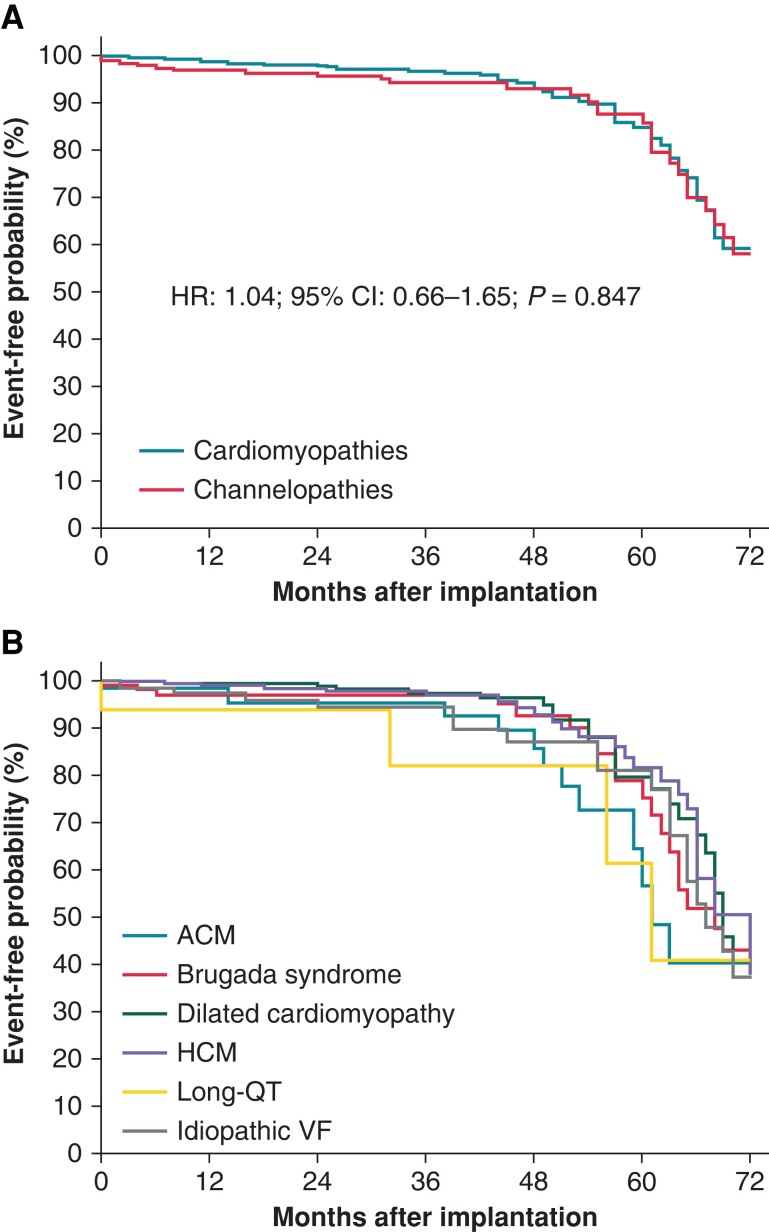
Kaplan–Meier estimates of time to first device-related complication stratified by cardiomypathies and channelopathies (*A*) and type of disease (*B*). ACM, arrhythmogenic cardiomyopathy; HCM, hypertrophic cardiomyopathy; VF, ventricular fibrillation.

Appropriate shocks were delivered in 38 (6%) patients during follow-up. The first shock was effective in 34 (90%) patients, and the final conversion rate was 97% (37 out of 38). Seventy-nine device-related complications were reported in 78 (12%) patients during follow-up. In the overall population, the rate of appropriate shocks and device-related complications at 12 months was 2.2% (95% CI: 1.2–3.7) and 1.8% (95% CI: 0.9–3.1), respectively. The rates of appropriate shock and device-related complications at 12 months according to the type of disease are reported in *Table 2*. Kaplan–Meier analysis of time to first appropriate shock, stratified by the type of cardiomyopathy/channelopathy, is shown in *Figure 3* (HR: 0.97; 95% CI: 0.47–2.02; *P* = 0.935). Kaplan–Meier analysis of time to first device-related complication is shown in *Figure 4* (HR: 1.04; 95% CI: 0.66–1.65; *P* = 0.847). The details of the events that occurred and their management are reported in *Table 3*. No sequelae were reported. The most common device-related complication was premature battery depletion requiring device replacement, which occurred in 50 (8%) patients. The need for cardiac resynchronization pacing or anti-bradycardia pacing was reported in five (0.8%) patients and required device explantation in four patients (see *Table 3* for details). No device explantation because of the need for anti-tachycardia pacing (ATP) was noted.

**Table 3:** Details of device-related complications reported during follow-up

Complications	Number	%	Management
Any complication	79	13%	
Pain or discomfort	2	0.3%	Surgical revision (*n* = 2)
Pocket-associated complications			
Erosion	3	0.5%	Surgical revision (*n* = 2), explantation (*n* = 1)
Infection	3	0.5%	Explantation (*n* = 3)
Lead-associated complications			
Lead dislodgment	2^a^	0.3%	Lead repositioning (*n* = 2)
Lead failure	4^a^	0.6%	Explantation (*n* = 4)
Lead infection	1	0.2%	Surgical revision (*n* = 1)
Inappropriate shock or sensing issue requiring system revision	7	1.4%	Explantation (*n* = 7)
Ineffective therapy requiring system revision	1	0.2%	Explantation (*n* = 1)
Battery advisory—early depletion	50	8.0%	Device replacement (*n* = 50)
Need for cardiac resynchronization pacing	2	0.3%	Explantation and transvenous CRT-D implantation (*n* = 2)
Need for anti-bradycardia pacing	3	0.5%	Explantation and transvenous ICD implantation (*n* = 2), leadless pacemaker implantation (*n* = 1)
**Additional events/clinical needs**			
Normal battery depletion	30	4.8%	Device replacement (*n* = 30)
Battery advisory—prophylactic replacement	11	1.8%	Device replacement (*n* = 11)
Heart transplantation	10	1.6%	Explantation (*n* = 10)
Ejection fraction improvement/indication re-evaluation	3	0.5%	Explantation (*n* = 3)
Ventricular tachycardia recurrences	3	0.5%	Catheter ablation (*n* = 3)

a In one case of lead dislodgement and in three cases of lead failure, patients also experienced inappropriate shocks.

## Discussion

In this multicentre study, we evaluated the rate of IS and device-related complications during long-term follow-up in patients with cardiomyopathies and channelopathies who had undergone S-ICD implantation. The main findings are:

1. Over a median follow-up of 43 months, IS occurred in 10% (3.5% at 12 months) of patients who received a modern device, with SMART Pass filter activated in most cases and high detection cut-off rates programmed. Non-cardiac oversensing was the leading cause of IS, suggesting the potentially positive effect of modern S-ICD in reducing IS due to TWOS.
2. The rate of IS at 12 months was higher in patients with cardiomyopathies than in those with channelopathies (4.6% vs. 1.1%), despite similar values over the entire follow-up period.
3. Patients with ACM presented the highest rate of IS at 12 months (10.9%).
4. Device-related complications were reported in 12% of patients (1.8% at 12 months). Pocket and lead complications were few (1% and 1.1%, respectively), while premature battery depletion was the most common (8%) complication. The management of all device-related complications requiring surgical intervention was safe and effective.
5. S-ICD was effective in terminating clinical VA, and we did not report a need for S-ICD explantation and TV-ICD reimplantation to ensure ATP therapy.
6. The need for anti-bradycardia pacing was low, occurring in 0.8% of patients.

Patients with cardiomyopathies and channelopathies constitute a heterogeneous group of patients at increased risk of SCD.^1–10^ The common denominators of patients with cardiomyopathies and channelopathies are younger age than the typical ICD population with coronary artery disease referred for primary or secondary prevention of SCD, no or minor comorbidities, and an active lifestyle.^1–11^ Young patients with cardiomyopathies and channelopathies have a predominantly arrhythmia-related prognosis and may survive for many decades and have nearly normal life expectancy thanks to the protection against SCD provided by the ICD,^1–11^ as confirmed by the low mortality rate observed in our study group (3% over a median follow-up of 43 months). In these patients, IS are frequently caused by either supraventricular tachycardia or abnormal sensing (due to T- or P-wave oversensing, lead fracture, or electromagnetic interference) and have the potential to reduce the patient’s quality of life and compromise the acceptance of ICD therapy. Accordingly, the risk/benefit ratio should be carefully assessed when considering ICD implantation for primary prevention, especially in patients with cardiomyopathies and channelopathies, and a high priority should be given to preventing IS by means of adequate device selection, targeted device programming, and modern discriminating software.^13,15–19,33–35^ In a large meta-analysis by Nordkamp et al.,^10^ which comprised 4916 young TV-ICD patients with inherited arrhythmia diseases (IADs), the crude annual IS rate was 4.7% per year. More recently, device manufacturers have implemented technological improvements in order to reduce inappropriate ICD interventions; this has resulted in more effective models of device programming and improved arrhythmia detection algorithms.^13,15–19^ Indeed, a systematic review and meta-analysis reported an annual IS rate of 6.4%, which later progressively decreased over time and significantly dropped to 1.9% in one of the more recent studies.^34^ Recently, Auricchio et al.^36^ conducted a subanalysis of the PainFree SST Study in which they specifically assessed the rate of IS in patients with IADs who had received TV-ICD endowed with the SmartShock Technology. The authors found that, in patients with IAD, the annualized IS rate was 1.6%. The study had some limitations, i.e. the small sample; the incomplete spectrum of IADs in analysis, which did not include important diseases such as HCM, dilated cardiomyopathy, catecholaminergic polymorphic VT, and short-QT syndrome; and the use of a TV-ICD from a single manufacturer. However, it confirmed the continuous improvement of detection algorithms, which may have a major impact on the patient’s quality of life, especially that of young IAD patients, who will have ICDs for a much longer time.

The most common complication of the TV-ICD is long-term transvenous lead issues; the S-ICD avoids such problems. Observational studies have shown the overall efficacy and safety of the S-ICD over medium- and long-term follow-up, despite relatively high IS rates. However, S-ICD therapy has evolved over the years, and more recent data show that the use of high-rate cut-offs, modern S-ICD with modern electrogram filtering, and discrimination algorithms has significantly reduced IS rates; indeed, with modern devices, the reported rate is 2.4%/year, a figure even lower than that of TV-ICD with modern programming.^13,15,17–19^ However, data on the performance of the S-ICD in patients with cardiomyopathies and channelopathies are scant, excluding observations on specific patient groups.^20–26^ Moreover, there are concerns regarding the presence of ECG depolarization/repolarization abnormalities, which may trigger IS, and the inability to deliver ATP, which may be an effective ‘pain-free’ therapy.

In a previous study by Rudic et al.,^22^ which involved patients with various IADs (24 with BrS, 17 with IVF, 6 with LQTS, 1 with short-QT syndrome, 3 with catecholaminergic polymorphic VT, 8 with HCM, and 3 with ACM) who had received an S-ICD, the IS rate was 3.2% over a median follow-up of 31.0 ± 14.2 months. In the Effortless study cohort of S-ICD patients with channelopathies, the incidence of IS was 8.5% over 3.2 years of follow-up and the annualized IS rate was lower among S-ICD patients than TV-ICD patients (2.7%/year vs. 3.8%year).^20^ In that study, most IS were caused by oversensing, principally cardiac oversensing (5.0%), including TWOS. In our patients with channelopathies, the IS rate was lower (1.1% at 12 months) than in the Effortless study,^20^ suggesting the potentially positive effect of the new-generation S-ICD in reducing IS due to TWOS.

A more recent study by Kuschyk et al.^21^ assessed the long-term outcome of S-ICD patients in comparison with TV-ICD patients in a cohort of patients with IADs; a relatively low incidence of IS (1.9%/year) was observed, and no statistically significant differences emerged between S-ICD and TV-ICD patients (though the rate was lower in the S-ICD group: 1.4%/year vs. 2.5%/year). These findings may be explained by the fact that modern devices were used in most S-ICD patients and improved programming strategies in both types of devices. The relatively higher rate of IS in our study may be explained by the difference between the study populations, in that ours had a higher prevalence of dilated cardiomyopathies, HCM, and, especially, ACM which is historically associated with higher rates of IS.^23,24,37^ Indeed, our patients with ACM had the highest rate of IS.

In their meta-analysis, Auricchio and co-workers^34^ found that studies with longer mean follow-up had lower annualized IS rates. It is likely that some patients are at higher risk of IS than others and tend to experience events earlier, leaving a lower risk group to the later follow-up times. In order to allow comparisons between groups, we estimated the IS rate at 12 months and observed a higher rate in patients with cardiomyopathies. This difference was not confirmed over the entire follow-up period, probably for the same reason. Indeed, patients with channelopathies might manifest IS later, owing to the evolution of the ECG signal detected by the device.

Regarding the causes of IS, non-cardiac oversensing was the leading cause in our population. This can certainly be explained by the SMART Pass filter of modern S-ICD, which attenuates cardiac oversensing and especially TWOS.^15,16,18^

Most IS in the present study were successfully managed by reprogramming the device, without system revision, confirming previous findings.^19^ The number of patients who experienced IS recurrences did not appear to be high in both groups. In particular, both first therapies and recurrences seemed infrequent in patients with channelopathies after the second year.

Overall, device-related complications that required revision were relatively infrequent, i.e. 12% over a median follow-up of 43 months (1.8% at 12 months), and were comparable among the different types of diseases. In comparison, the rate of device-related complications at 1 year was ∼4% among the S-ICDs in the PRAETORIAN study.^12^ Early battery depletion was the most frequent cause of system revision in our study. Indeed, in 2020, the EMBLEM S-ICD was subjected to a safety notification because of an increased risk of rapid battery depletion (Boston Scientific urgent field action REF.92400926-FA). In addition, 4.8% of the devices underwent normal battery depletion, being replaced after about 6–7 years, depending on the number of therapies delivered and in line with the generator projected longevity. The rates of pocket and lead complications were low. This can be ascribed to the fact that the pulse generator was most often implanted in the intermuscular space rather than in the traditional subcutaneous pocket and the two-incision technique was used for lead deployment.^31,32^ Indeed, these approaches have recently been shown to result in fewer device-related complications and composite endpoints of complication or IS over medium-term follow-up.^32^

Like other investigators,^20,21,23^ we observed a relatively low rate of appropriate shocks in S-ICD patients with cardiomyopathy and channelopathies. The high-rate cut-off programmed in S-ICDs and the multistep discriminative sensing algorithm may have reduced therapies, thus allowing many VA to self-terminate while still protecting against life-threatening VT/VF. Indeed, in previous studies, appropriate ICD intervention rates were lower in the S-ICD group than in the TV-ICD group, with no reduction in overall efficacy.^20,21,23,38^

The main potential limitation of the S-ICD is its inability to deliver ATP, which may be an effective ‘pain-free’ therapy in patients with structural heart diseases. In our study, no patients had the device removed because of a perceived need for ATP. Indeed, in TV-ICD patients with cardiomyopathies and channelopathies, the availability of ATP therapy is not reported to result in fewer appropriate shocks.^21^ This may be explained by the fact that the majority of appropriate shocks in patients with cardiomyopathies and channelopathies is triggered by fast or polymorphic VT, which are less amenable to ATP.^21^ Thus, the decision of whether to implant an S-ICD in patients with structural cardiomyopathies needs to be patient-specific; indeed, the probability of lead-related complications, which are typically observed in TV-ICD patients, must be balanced with the likelihood of recurrent VT, which may be effectively pace-terminated.

Possible strategies that could further reduce IS in patients with channelopathies and cardiomyopathies who receive modern S-ICDs are (i) thorough pre-implantation ECG screening and device programming with a high-rate cut-off (shock zone >250); (ii) targeting a surface ECG R-wave amplitude >1 mV and an appropriately high R/T and R/P amplitude ratio on implantation, which may allow better discrimination;^39^ (iii) tracking the sensed R-wave amplitude in various vectors at rest, during effort or Ajmaline challenge in BrS patients;^40,41^ (iv) considering patients with at least two suitable vectors, as the potential decline in R-wave amplitude during follow-up, especially in ACM patients, increases the risk of cardiac and/or non-cardiac oversensing;^39^ and (v) performing provocative tests at follow-up visit aiming for myopotential inducibility to select the optimal sensing vector.^42^

### Limitations

The limitations of our study should be acknowledged. First, its observational and retrospective design may have introduced an inherent bias. Second, the small size of some groups did not allow us to accurately estimate the rate of endpoints or make direct comparison between groups. No direct comparison was made between TV-ICD and S-ICD nor between the traditional technique of S-ICD implantation and the intermuscular two-incision technique; however, this goes beyond the aim of the present study. Clearly, large randomized studies involving a predefined comparable cohort of patients with new-generation TV-ICDs endowed with updated discrimination algorithms and software would be needed in order to accurately define the clinical benefit or harm resulting from device choice. Despite our long follow-up, a relatively small number of events occurred, and this might have prevented us from identifying predictors. Finally, as all our procedures were performed by experienced operators, the results may not be widely applicable in less experienced centres. Despite these limitations, the data presented are unique in several ways and make an important contribution to the scant published data regarding the clinical performance of modern S-ICDs in patients with channelopathies and cardiomyopathies.

## Conclusions

According to our findings, modern S-ICDs may be a valuable alternative to TV-ICDs in patients with cardiomyopathies and channelopathies who do not need anti-bradycardia pacing or ATP therapy. However, the potential risk of IS, mainly due to non-cardiac oversensing, should be considered. Our findings suggest the potential positive effect of modern S-ICD in reducing IS due to TWOS. Strategies for avoiding IS must be adopted.

## Supplementary Material

euad239_Supplementary_Data

## Data Availability

The experimental data used to support the findings of this study are available from the corresponding author upon request.

## Appendix

List of participating centres

- ASST Rhodense, Rho-Garbagnate Milanese, Milan: G.L. Botto, F.L. Canevese, and M.C. Casale
- ASST Sette Laghi, Ospedale di Circolo e Fondazione Macchi, Varese: F. Caravati
- Azienda Ospedaliera ‘G. Brotzu’, Cagliari: B. Schintu, A. Scalone, G. Tola, and A. Setzu
- Azienda Ospedaliera Mater Domini, Catanzaro: A. Curcio
- Azienda Ospedaliera Universitaria Senese, Siena: A. Santoro, C. Baiocchi, R. Gentilini, and S. Lunghetti
- Clinica Montevergine, Mercogliano, Avellino: F. Solimene, G. Shopova, V. Schillaci, A. Arestia, and A. Agresta
- Fatebenefratelli Hospital, Rome: S. Bianchi, P. Rossi, and F. M. Cauti
- Fondazione Poliambulanza, Brescia: C. La Greca and D. Pecora
- ‘Giovan Battista Grassi’ Hospital, Ostia, Rome: F. Ammirati, L. Santini, K. Mahfouz, and C. Colaiaco
- IRCCS Fondazione Policlinico ‘S. Matteo’, Pavia: R. Rordorf, A. Vicentini, S. Savastano, B. Petracci, A. Sanzo, E. Baldi, and M. Casula
- Istituto Auxologico Italiano—IRCCS, Milan: GB. Perego and V. Rella
- Istituto Clinico Sant'Ambrogio, Milan: L. Ottaviano
- Monaldi Hospital, Naples: A. D’Onofrio, V. Bianchi, V. Tavoletta, and S. De Vivo
- Ospedale ‘G. Panico’, Tricase, Lecce: P. Palmisano and M. Accogli
- Ospedale ‘Vito Fazzi’, Lecce: E. Pisanò and G. Milanese
- Ospedale Carlo Poma, Mantova: P. Pepi and D. Nicolis
- Ospedale di Legnano, Milan: M. Mariani and M. Pagani;
- Ospedale Di Venere, Carbonara di Bari, Bari: Massimo Vincenzo Bonfantino
- Ospedale F. Miulli, Acquaviva delle Fonti, Bari: V. Caccavo, M. Grimaldi, and G. Katsouras
- Ospedale Luigi Sacco, Milan: GB. Forleo
- Ospedale Maggiore, Crema: E. Chieffo and E. Tavarelli
- Ospedale Manzoni, Lecco: R. Brambilla and A. Pani
- Ospedale Maria Vittoria, Turin: M. Giammaria, M.T. Lucciola, and C. Amellone
- Ospedale Melorio, Santa Maria Capua Vetere, Caserta: C. Uran
- Ospedale Niguarda Cà Granda, Milano: M. Baroni
- Ospedale Papa Giovanni XXIII, Bergamo: P. De Filippo, P. Ferrari, and C. Leidi
- Ospedale Pediatrico ‘Bambino Gesù’, Palidoro, Fiumicino: F. Drago, M.S. Silvetti, V. Pazzano, S. Russo, R. Remoli, I. Battipaglia, I. Cazzoli, and F. Saputo
- Ospedale S. Andrea, La Spezia: C. Devecchi
- Ospedale S. Andrea, Vercelli: L. Barbonaglia
- Ospedale S. Anna e S. Sebastiano, Caserta: M. Viscusi, M. Brignoli, and A. Mattera
- Ospedale S. Anna, Como: S. Pedretti
- Ospedale S. Biagio, Domodossola: A Lupi and S. Tommasi
- Ospedale S. Camillo de Lellis, Rieti: A. Kol, M. C. Gatto, and A. Persi
- Ospedale S. Croce e Carle, Cuneo: A. Gonella, G. Rossetti, E. Menardi, and R. Rossini
- Ospedale S. Donato, Arezzo: P. Notarstefano, M. Nesti, and A. Fraticelli
- Ospedale S. Maria, Terni: G. Carreras, S. Donzelli, C. Marini, A. Tordini, and L. Lazzari
- Ospedale S. Martino, Genova: P. Sartori, P. Rossi, P. Di Donna, and G. Mascia
- Ospedale San Giovanni Bosco, Naples: P. Capogrosso, P. Magliano, and M. Colimodio
- Ospedale San Raffaele, Milan: S. Sala, P. Mazzone, and P. Della Bella
- Ospedale SS. Annunziata, Savigliano, Cuneo: A. Coppolino
- Ospedale SS. Giacomo e Cristoforo, Massa: G. Arena, V. Borrello, M. Ratti, and C. Bartoli
- Ospedale S. Andrea, Rome: P. Francia, F. Palano, and C. Adduci
- Ospedale Villa Scassi, Genova: A. Torriglia and M. Laffi
- Ospedali Riuniti San Giovanni di Dio e Ruggi d'Aragona, Salerno: M. Manzo, C. Esposito, A. Giano, and F. Franculli
- Ospedali Riuniti, Reggio Calabria: A. Pangallo
- P.O. Ferrari, Castrovillari, Cosenza: G. Bisignani and S. De Bonis
- Paediatric Cardiology Unit, Second University of Naples, Napoli: B. Sarubbi, D. Colonna, A. Correra, and E. Romeo
- Policlinico Federico II, Naples: A. Rapacciuolo, V. Liguori, A. Viggiano, and T. Strisciullo
- Policlinico S.Orsola-Malpighi, Bologna: M. Biffi, I. Diemberger, M. Ziacchi, and C. Martignani
- Policlinico Umberto I, Rome: A. Piro, C. Lavalle, M. Magnocavallo, and M. V. Mariani
- Policlinico Universitario Campus Bio-Medico, Rome: D. Ricciardi, V. Calabrese, F. Gioia, and F. Picarelli
- Presidio Ospedaliero Muscatello, Augusta (SR): G. Licciardello and G. Busacca
- Presidio Ospedaliero Rodolico, Catania: V.I. Calvi
- ‘San Eugenio’ Hospital, Rome: F. Lamberti, G. Lumia, C. Bellini, and C. Bianchi
- ‘San Giovanni Battista’ Hospital, Foligno: G. Savarese, C. Andreoli, L. Pimpinicchio, and D. Pellegrini
- ‘San Luca’ Hospital, Lucca: D. Giorgi, Bovenzi, and F. Busoni
- Santa Maria della Misericordia, Udine: E. Daleffe, D. Facchin, and L. Rebellato
- Università di Tor Vergata, Rome: G. Stifano, G. Magliano, D. Sergi, L. Barone, and R. Morgagni
- Università Politecnica delle Marche, Ancona: A. Dello Russo, M. Casella, F. Guerra, L. Cipolletta, and S. Molini
- University Hospital of Pisa, Pisa: R. De Lucia, A. Di Cori, G. Grifoni, L. Paperini, L. Segreti, E. Soldati, S. Viani, and G. Zucchelli
- University of Campania ‘Luigi Vanvitelli’, Naples: G. Nigro, V. Russo, A. Rago, E. Ammendola, and A. Papa
- University of Florence, Florence: P. Pieragnoli, G. Ricciardi, L. Checchi, and L. Perrotta
- University of Padua: F. Migliore
