# Supplementary figures and images for: Modern subcutaneous implantable defibrillator therapy in patients with cardiomyopathies and channelopathies: data from a large multicentre registry
Source: Europace. 2023 Aug 3;25(9):euad239. doi: 10.1093/europace/euad239 (PMC10438213; doi:10.1093/europace/euad239)

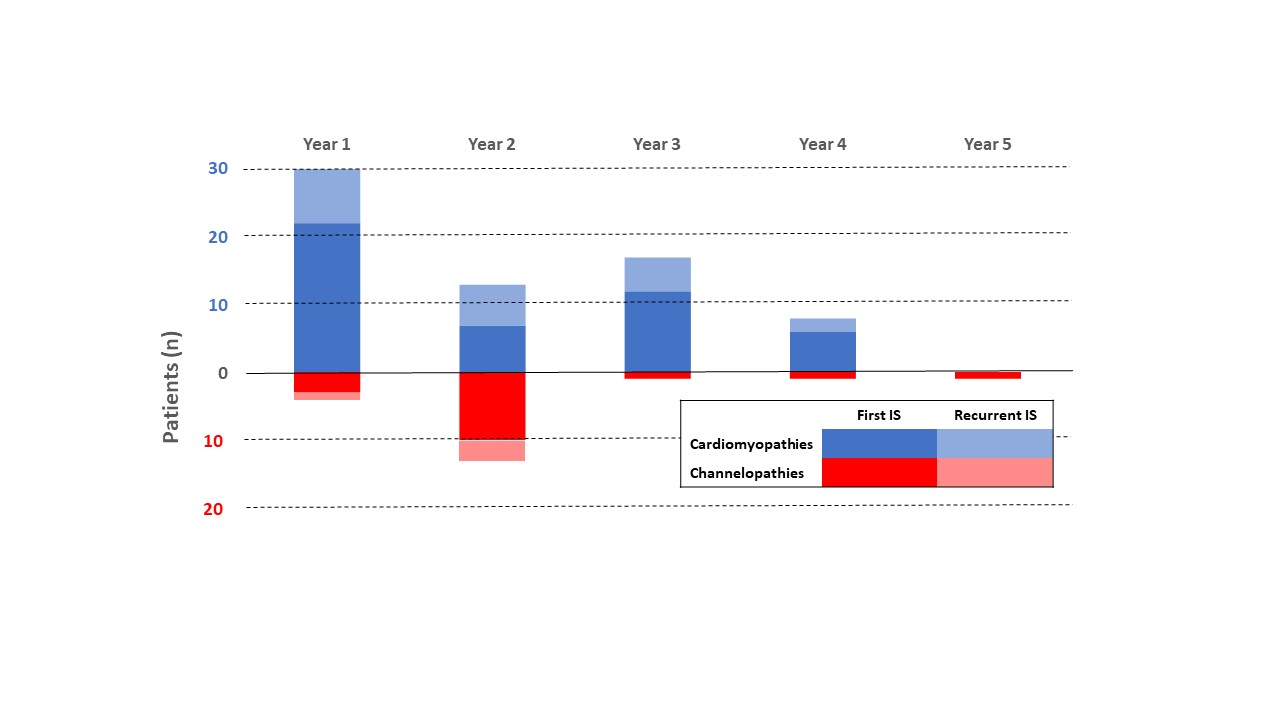

Supplement: euad239_Supplementary_Data [file euad239_supplementary_data.jpeg]
